# A Concept Analysis of Attitude toward Getting Vaccinated against Human Papillomavirus

**DOI:** 10.1155/2013/373805

**Published:** 2013-05-28

**Authors:** Nop T. Ratanasiripong, Kathleen T. Chai

**Affiliations:** School of Nursing, California State University, Dominguez Hills, CA 90747, USA

## Abstract

In the research literature, the concept of attitude has been used and presented widely. However, attitude has been inconsistently defined and measured in various terms. This paper presents a concept analysis, using the Wilsonian methods modified by Walker and Avant (2004), to define and clarify the concept of attitude in order to provide an operationalized definition for a research study on attitudes toward a behavior: getting vaccinated against HPV. While the finding is not conclusive, three attributes of attitude: belief, affection, and evaluation are described. A theoretical definition and sample cases are constructed to illustrate the concept further. Antecedents, consequences, and empirical referents are discussed. Recommendations regarding the use of the concept of attitude in research, nursing practice, and nursing education are also made.

## 1. Introduction

The concept of “attitude” has been used widely in daily living communication and in science. As of the end of 2012, when entering the term “attitude” as a keyword in the “title search” on the Medline and PubMed databases for biomedical and life science publications, over 6,400 articles have been identified on each database. On Cumulative Index to Nursing and Allied Health Care Literature (CINAHL), there are 2,041 articles with the word attitude as a part of the title. In the nursing discipline, nurse scholars have used the attitude concept in conducting research studies in various populations including nursing staff, nursing students, and patients or clients (EBSCO Publishing, 2012). Nurses may use / refer to the term “attitude” as a part of patient assessment or explaining a patient's character. Although the concept of attitude (toward an object, person, thing, issue, event, and behavior) has been commonly used in the nursing literature, the abstract concept of attitude has not been clearly defined [1, 2].

In the beginning steps of developing a research study, it is important that the concepts of the study are reviewed, defined, and provided operational definitions. “Concept” is a major component of theory and conveys the abstract ideas within theory [3]. Concept provides us with a concise summary of thoughts related to a phenomenon or a group of phenomena [4]. The nursing discipline has traditionally valued the concept analysis process to help identify appropriate terms to use in subsequent research and as a means to determine the suitable methodology for investigating the concept of interest [5]. Walker and Avant (2004) describe concept analysis as a process of examining the basic elements of a concept [6]. The analysis process allows us to distinguish similar concepts and the similarity and differences between concepts. It also helps simplify, clarify, refine, and determine the concept's internal structure. 

This paper aims to clarify the concept of “attitude” to facilitate the development of a research study of attitudes toward a behavior: obtaining the human papillomavirus (HPV) vaccine. However, the authors also believe that this analysis may be beneficial for other nurse scholars who consider examining the attitude concept in other behavioral aspects.

## 2. Materials and Methods

In the analysis of the concept of attitude toward a behavior, the Wilsonian methods of concept analysis modified by Walker and Avant are used [6]. Walker and Avant were among the first to bring Wilson's techniques of concept analysis into nursing [7]. They modified Wilson's 11 techniques into eight steps for beginners to use. Even though this method is criticized for its lack of the intellectual rigor, the method is easy to use [8] and seems to be very appropriate for novice researchers by providing a structural guideline to conduct a concept analysis. The concept analysis steps used in this paper are as follows: (1) selecting a concept, (2) determining the aims or purposes of analysis, (3) identifying all uses of the concept that can be discovered, (4) determining the defining attributes, (5) identifying a model case, (6) identifying borderline, related, invented, contrary, or illegitimate cases, (7) identifying antecedents and consequences, and (8) defining empirical referents [6].

## 3. Results and Discussion

### 3.1. Uses of the Concept

As the literature is being used as data in the analysis of a concept, adequacy and appropriateness of the chosen sample are important evaluative criteria [9]. This literature review methodology includes reviewing definitions of “attitude” in various dictionaries, searching through “Google” by using attitude as a keyword, reading attitude-related theories from various textbooks, and reviewing research abstracts or articles found on PubMed, Medline, and CINAHL. The findings from the literature review are discussed in the following section.

#### 3.1.1. General Definitions of Attitude

The term “attitude” is a French term, originated from the Italian word “attitudine” and originally from the late Latin “aptitudinis” and “aptitude” [10]. An internet search for the “attitude” term by using the Google search engine resulted in over 340 million citations in various websites such as medical/health education, psychosocial test, quotes, magazine, movie/music entertainment, and aviation. The term “attitude” is most often defined or referred to as a noun. Based on Webster's New World Dictionary of the American Language [10], the definitions of attitude are

1. the position or posture assumed by the body in connection with an action, feeling, mood, and so forth (to kneel in attitude of prayer);
2. a manner of acting, feeling, or thinking that shows one's disposition, opinion, and so forth (a friendly attitude);
3. one's disposition, opinion, mental set, and so forth;
4. the position of an aircraft or spacecraft in relation to a given line or plane, as the horizon.

The following definitions are also found in various dictionaries. The American Heritage Dictionary of the English Language [11] offers the following. (1a) A state of mind or a feeling; disposition. (1b) An arrogant or hostile state of mind or disposition. (2) A position of the body or manner of carrying oneself. See synonyms at posture. (3) The orientation of an aircraft's axes relative to a reference line or plane, such as the horizon. (4) The orientation of a spacecraft relative to its direction of motion. (5) A position similar to an arabesque in which a ballet dancer stands on one leg with the other raised either in front or in back and bent at the knee. Merriam-Webster [12] supplies the following. (1a) A mental position with regard to a fact or state (a helpful attitude). (1b) A feeling or emotion toward a fact or state. (2) The arrangement of the parts of a body or figure: posture. (3) A position assumed for a specific purpose (a threatening attitude). (4) A ballet position similar to the arabesque in which the raised leg is bent at the knee. (5) The position of an aircraft or spacecraft determined by the relationship between its axes and a reference datum (as the horizon or a particular star). (6) An organismic state of readiness to respond in a characteristic way to a stimulus (as an object, concept, or situation). (7a) A negative or hostile state of mind. (7b) “A cool, cocky, defiant, or arrogant manner” and “predisposition or a tendency to respond positively or negatively towards a certain idea, object, person, or situation.” The Business Dictionary adds “attitude influences an individual's choice of action and responses to challenges, incentives, and rewards” [13]. 

From the definitions described in dictionaries and thesauri, the definitions related to physical and aircraft positions are omitted from analysis as they are not pertinent to the psychosocial use and these definitions are out of the authors' research interest context. With those definitions being excluded, the remaining definitions of attitude seem to be within the research context and these definitions are repeatedly mentioned in similar ways. Those meanings involve the position of feeling or affection, thinking, or acting that shows one's belief and/or opinion.

#### 3.1.2. Theoretical Definitions of Attitude

In the psychosocial discipline, the attitude concept received its first attention from Darwin in 1872. Darwin defined attitude as a motoric (behavioral) concept or physical expression of an emotion [14]. In the 1930s, psychologists agreed that all attitudes contain an evaluative component. They also viewed that attitude has both affective and belief components and that attitude and behavior should be consistent. “Attitude toward a behavior” is constructed by beliefs about engaging in the behavior and the associated evaluation of that belief [14]. Crano and Prislin viewed attitude as the evaluative judgment that integrates and summarizes cognitive and affective reactions [15]. 

 According to the theory of planned behavior, if behavior is under volitional control, the intention to perform an action will highly correlate with the action itself [16]. The theory refers to “attitude toward the behavior” as “the degree to which a person has favorable or unfavorable evaluation or appraisal of the behavior in question” [16]. Attitudes are made up of the beliefs people hold about the object and the associated evaluation of that belief. The theory posits that attitude is usually assumed to form a bipolar continuum, from a negative evaluation on one end to a positive evaluation on the other [16]. 

Another model related to attitude is the attitude accessibility theory. The theory defines attitude as a learned association between a concept and an evaluation [17]. The theory indicates that the more rapidly an attitude can be expressed, the greater its strength. The stronger the attitude, the more accessible it is. Highly accessible attitudes are more difficult to change. Attitudes that are highly accessible from memory are more likely to guide behavior than less accessible attitudes [17]. 

From selected psychosocial theory perspectives above, the attitude concept is described as evaluation or appraisal of an object/issue/behavior after a person learns or acquires the information about the object/issue/behavior. How a person evaluates the issue may depend on what the person believes. The personal evaluation may result in a bipolar continuum of attitude. The attitude is then demonstrated through a physical or emotional expression. Attitude also can lead to behavioral intention and action. 

#### 3.1.3. Research Definitions of Attitude

The literature searches in PubMed were conducted with the keyword “attitude.” There were over 250,000 articles shown. To narrow down the finding, “HPV” keyword was added and the publication year was limited to five years. Out of 527 articles shown, 70 research studies indicated the attitude term on the titles. However, 52 studies conducted outside the United States were excluded due to the assumption that the attitude concept might be defined, understood, and utilized differently in various cultures and languages, leaving 17 studies for further review. After reviewing the abstracts, seven studies with attitudes related to HPV vaccination or getting vaccinated against HPV were chosen for the review.

Out of these seven studies, five studies included the term “attitude” as a part of their study aims or objectives [18–22]. One study stated the study objective as to “identify psychosocial factors correlated to HPV vaccination intention” [23] and another study used the word vaccine “acceptability” instead of attitude as a part of the study objective [24]. Six studies quantitatively measured the attitude concept by using a questionnaire [18–20, 22–24]. Out of these, one study measured the attitude concept by asking the participants what they thought about HPV vaccination, using a semantic differential scale (i.e., harmful/beneficial, bad/good). However, the definition of attitude is not specifically presented [23]. The attitude toward HPV vaccine or vaccination was interchangeably referred to and expressed through the verbs such as want, desire, feel, concern, accept, and support [18–20, 22, 24] and through the nouns such as belief, opinion, and willingness [19, 22, 23]. One study qualitatively explored attitude toward the vaccine by using a semistructure interview [21]. The study reported that the participants were “positive about the HPV vaccine” and often used the words such as belief, view, thinking, risk determination perception, and acceptance in order to present the attitude concept [21].

The literature review of attitude-related studies obviously showed that the definitions of attitude are not directly presented and often measured under various terms or concepts such as belief, feeling/desire, opinion, concern, and acceptance. However, the reviewed articles showed one common theme of referring to attitude with the spectrum from positive to negative. 

### 3.2. Working Definition and Defining Attributes

Walker and Avant stated that determining the defining attribute is the most critical part of concept analysis [6]. Defining the attributes can be done by searching for the cluster of attributes that are the most frequently associated with the concept. When analyzing the findings from various perspectives, the authors somewhat agree with Altmann's conclusion that attitude has cognitive and affective components [1]. However, the authors believe that a behavioral component is not an attribute, but a consequence of attitude. The authors propose three characteristics that have repeatedly appeared or been used to describe the concept of attitude: belief, feeling, and evaluation.

Based on the above defining attributes of attitude, it is possible to theoretically define attitude toward a behavior as a feeling response toward the behavior once the person has evaluated the behavior based on the person's belief. The attitude can be physically or verbally expressed in a negative to positive continuum. In addition, because the attitude concept is sometimes used interchangeably with the “opinion” concept in the literature review, it is important to differentiate these two concepts to confirm that the attitude concept's definition and attributes are properly established. 

 “Opinion” is described as “(1) a belief not based on absolute certainty or positive knowledge but on what seems true, valid, or probable to one's own mind, judgment; (2) an evaluation, impression, or estimation of the quality or worth of a person or thing; (3) the formal judgment of an expert on a matter in which his advice is sought; (4) law, the formal statement by a judge, court referee, and so forth of the law bearing on a case” [10]. The authors believe that the opinion concept may have shared belief and evaluation attributes with attitude concept. However, opinion may not contain the feeling attribute.

### 3.3. Antecedents

 Walker and Avant [6] posited that identifying antecedents and consequences is helpful in further refining the defining attributes. Antecedents are events that must take place before the attitude concept. A defining attribute cannot be either an antecedent or a consequence. 

In the case of attitude concept, attitude theorists believe that the attitude is acquired through cognitive learning experiences [15, 25]. Past experiences and informational influences contribute to the attitude at the time it occurs. Attitude is the result of sequential and information integration processes. Altmann [1] provided an example of a child who was born with no past experiences and free from ideas. After gathering information and gaining more life experiences, the child then developed his/her attitude. In the authors' own words, attitude required ability to think and make a decision. Attitude develops over years when the person acquires more knowledge and experience in life. Attitude can be influenced by culture and can be changed through time and space.

### 3.4. Consequences

Consequences are the incident or events that happen as a result of the occurrence of the concept [6]. Based on theory of reasoned action and theory of planned behaviors, attitude toward a certain behavior is a strong predictor of intention to perform that specific behavior [2, 16]. Attitude is also a good prediction of behavior [2, 16, 17]. Altmann [1] noted that, while people do not always act according to an attitude, they are predisposed to behave in a certain manner. In summary, the authors believe that the consequences of attitude are behavioral intention, maintenance, or change, which in turn, can result either in positive or negative health outcomes.

### 3.5. Cases

Identifying a model case and additional cases help define and refine the attributes of the concept [6]. The following cases are created in order to find the best fit attributes. 

#### 3.5.1. Model Case

A female college student attends a sexual health workshop and learns about HPV and HPV vaccine. She believes that getting the HPV vaccine and using safe sexual practices will be beneficial to prevent HPV. She desires to obtain the vaccine and decides to do so. This model case illustrates a real life example and demonstrates all the defining attributes: belief, feeling, and evaluation of the attitude concept.

#### 3.5.2. Borderline Case

A teenage girl starts having sexual intercourse with her boyfriend. When the mother of the girl knows about this, she decides to talk about sexually transmitted infections (STIs) and HPV vaccination. The girl then decides to get an HPV vaccine for her mother's “peace of mind.” This example is considered as a borderline case because it contains most of the attributes of the attitude concept but not all of them. In this case, the girl may believe that HPV vaccine protects against cervical cancer and is safe. Nevertheless, she may not favor the idea of getting vaccinated (feeling). Her decision to obtain the vaccine is because of her mother not because how she believes, evaluates, and feels about getting vaccinated against HPV.

#### 3.5.3. Related Case

A nursing student studies about HPV and other STIs for her final examination. She memorizes the cause and consequences of each STI. Then, she takes the test. She is glad that she passes the examination. This related case example shows a knowledge or cognition concept that is related to the attitude concept but does not contain all the defining attributes of attitude.

#### 3.5.4. Contrary Case

A baby is in a pediatrician office's waiting room for an annual checkup. There are posters about HPV vaccine posted on the wall. The baby is smiling when looking at the bright colorful posters. This case clearly demonstrates what the attitude concept is not. This case does not meet any criteria of attitude attributes. The baby has not gained a set of beliefs to evaluate the vaccine. The baby's smile is not related to HPV issue but to the colorful pictures.

#### 3.5.5. Illegitimate Case

At a theater, many young women are practicing standing on one leg with the other raised either in front or in back and bent at the knee. They try to make their attitudes balanced and perfectly synchronous. This case example demonstrates the attitude concept term entirely used out of the authors' research context.

### 3.6. Empirical Referents

 Empirical referents are “classes or categories of actual phenomena that by their existence or presence demonstrate the occurrence of the concept itself” [6, Page 46]. Empirical referents are very useful in instrument development because they are clearly linked to the theoretical base of the concept, thus contributing to both the content and construct validity of the instrument. Since attitude cannot be measured directly, measuring attitude may be more appropriately done by correlating and combining the findings from defined attributes of attitude. This method may more accurately support the inferences made regarding an attitude [1].

 Since the authors are planning to conduct a cross sectional survey, observation of “attitude” through behaviors or actions (e.g., talking positively about obtaining the vaccine) will not be included. The expression of attitude will be measured by asking the research participants to complete an attitude survey toward getting vaccinated against HPV. The attitude questionnaire will be built by combining the attitude attributes of belief, evaluation, feeling, and bipolarity into the questions. 

### 3.7. Discussion

In the process of reviewing the current literature, the attitude concept has been inconsistently defined and measured in various terms (i.e., belief, opinion, acceptance, and perception). When thinking about the model and additional cases, the authors realized that it was difficult to create true borderline and related cases in order to differentiate the attitude concept from the opinion concept. It seemed that the scopes of these concepts are overlapping. These findings confirmed that the attitude concept is indeed very abstract and may not be directly measured. While there is no current solution to measure the attitude more clearly, the authors suggest that researchers interested in measuring the attitude concept should consider building a research tool based on belief, feeling, and evaluation as the attributes of the concept. In addition, while opinion may be assessed by using “agree-disagree” questions, attitude may be assessed by using “desirable-undesirable, valuable-worthless, pleasant-unpleasant, interesting-boring, positive-negative, good-bad, like-dislike” questions which show the affection and evaluation attributes in a bipolar spectrum [2, 16]. Once the tool is tested and used, additional concept reviews and revisions of the tool may be necessary to improve attitude concept measurement.

## 4. Conclusion and Recommendations

The authors' philosophical belief toward getting to the truth is to seek an answer from one perspective and then seek answers from various perspectives. Although the authors may not be able to get the truth/answer of the real meaning of “attitude,” they believe they can come close to it. In order to do so, more research studies can be conducted to test and refine the attitude definition and attributes. For nursing practice, the attitude concept can be continually used as a part of patient assessment. As described above, attitude can influence behavior. Nurses may understand patient's particular behaviors when they also assess and are aware of certain attitudes toward the behavior in question. Nurses can also create an intervention focusing specifically on changing attitude to influence changing such behavior. Finally, in nursing education, while nursing organizations have suggested that nursing programs need to assess not only knowledge and competency but also attitude in nursing students, the measurement of attitude is not clear and is not standardized [26]. To ensure that conducting an attitude assessment in nursing students is useful, nursing organizations should define the attitude concept, tailor, and standardize the attitude assessment tool that can be used across the board. Otherwise, having attitude as a part of the program assessment may not be useful for benchmarking results.
